# Andean surface uplift constrained by radiogenic isotopes of arc lavas

**DOI:** 10.1038/s41467-018-03173-4

**Published:** 2018-03-06

**Authors:** Erin M. Scott, Mark B. Allen, Colin G. Macpherson, Ken J. W. McCaffrey, Jon P. Davidson, Christopher Saville, Mihai N. Ducea

**Affiliations:** 1 Department of Earth Sciences, Durham University, Durham, DH1 3LE UK; 2 Department of Geosciences, University of Arizona, Tucson, AZ 85721 USA; 3 Faculty of Geology and Geophysics, University of Bucharest, 010041 Bucharest, Romania

## Abstract

Climate and tectonics have complex feedback systems which are difficult to resolve and remain controversial. Here we propose a new climate-independent approach to constrain regional Andean surface uplift. ^87^Sr/^86^Sr and ^143^Nd/^144^Nd ratios of Quaternary frontal-arc lavas from the Andean Plateau are distinctly crustal (>0.705 and <0.5125, respectively) compared to non-plateau arc lavas, which we identify as a plateau discriminant. Strong linear correlations exist between smoothed elevation and ^87^Sr/^86^Sr (*R*^2^ = 0.858, *n* = 17) and ^143^Nd/^144^Nd (*R*^2^ = 0.919, *n* = 16) ratios of non-plateau arc lavas. These relationships are used to constrain 200 Myr of surface uplift history for the Western Cordillera (present elevation 4200 ± 516 m). Between 16 and 26°S, Miocene to recent arc lavas have comparable isotopic signatures, which we infer indicates that current elevations were attained in the Western Cordillera from 23 Ma. From 23–10 Ma, surface uplift gradually propagated southwards by ~400 km.

## Abstract (web summary)

Multiple complex tectonic and climatic processes have formed the Andes, which today provides a unique ecological niche. Here, Scott *et al*. investigate how the chemical composition of lavas from stratovolcanoes can be used to give insight on the uplift of the Andes over the last 200 million years.

## Introduction

Orogenic plateaux have complex tectonics and variable climates, which provide a unique ecological niche. Knowledge of the tectonic evolution and surface uplift of such high, wide regions is fundamental to understanding feedbacks between climate change and tectonics^1,2^. Orogenic plateaux affect atmospheric circulation and precipitation patterns^3^. Uplift of high plateaux changes the efficiency of erosion and sediment flux into internal and oceanic basins, leading to atmospheric CO_2_ drawdown via silicate weathering and hence long-term global climate cooling^2,3^. Conversely, arcs erupted through high plateaux emit large quantities of CO_2_ during magmatic flare-ups, which have been linked to global greenhouse events^4^. Climate-driven aridification and subsequent trench sediment starvation can also focus plate boundary stresses at subduction zones and enhance compressional deformation^1^. Despite numerous multidisciplinary studies the topographic, tectonic and geodynamic evolution of orogenic plateaux remains ambiguous^1,5–7^.

**Fig. 1:**
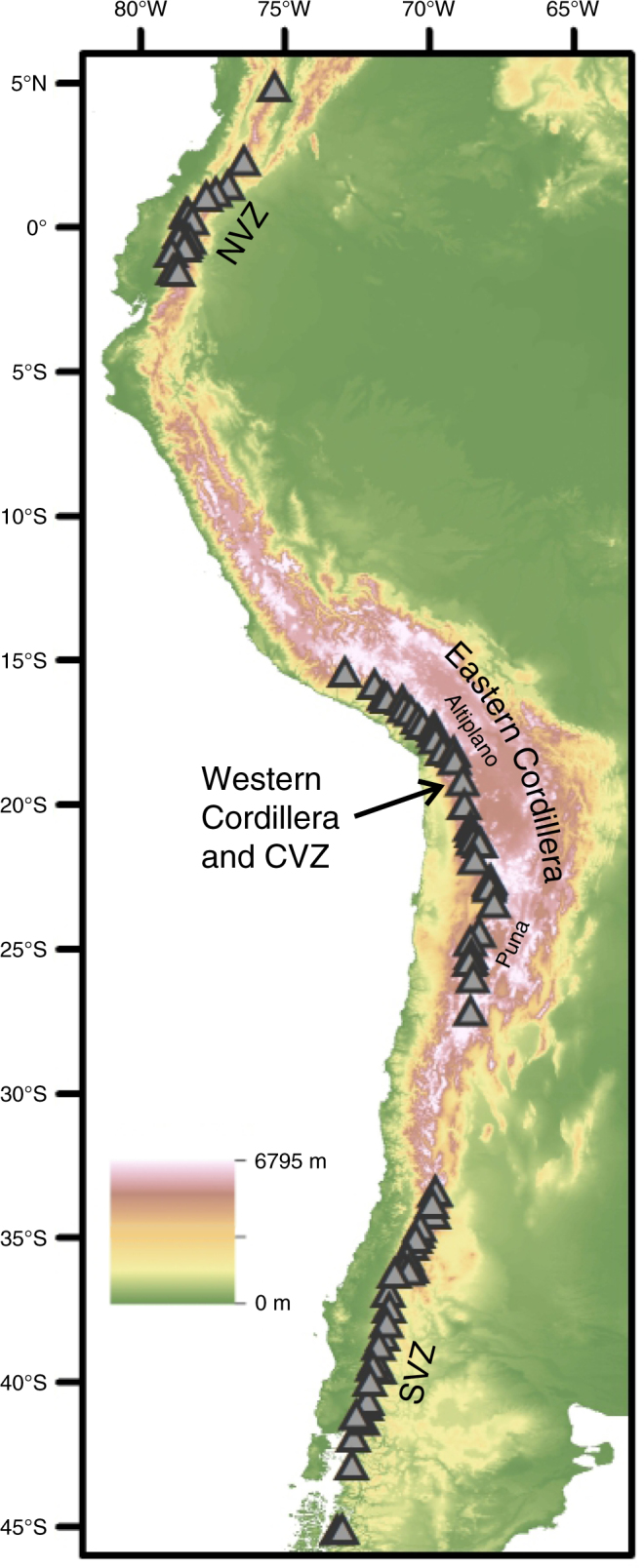
Topographic map of western South America. Grey triangles are locations of Quaternary arc front volcanoes from the NVZ, CVZ and SVZ (North, Central and Southern Volcanic Zones) included in our geochemical compilation (Methods). CVZ centres are located within the Western Cordillera of the Andean Plateau

The Andean Plateau is the second largest tectonically active plateau in the world. From west to east the Andean Plateau spans over 400 km and is divided into three tectonically distinct zones: the Western Cordillera (including the active Central Volcanic Zone, CVZ, of the Andean arc); the internally drained Altiplano and Puna plateaux; and the Eastern Cordillera fold-and-thrust belt (Fig. 1). North and south of the plateau Andean arc magmatism continues in the Northern and Southern Volcanic Zones (NVZ and SVZ, respectively), which are separated from the CVZ by two volcanic gaps. Large volumes of Andean arc magmatism have been emplaced along the South American margin since >200 Ma as result of oceanic subduction under the South American continent^8^. During this time the locus of Central Andean arc magmatism has progressively shifted eastward from the modern coastline to its present location^8,9^.

Many studies have attempted to quantify Andean Plateau surface uplift but most of these works concentrate on regions east of the active arc in the Altiplano, Puna and Eastern Cordillera^6^. Two end-member models of Andean Plateau uplift remain prevalent^5,6^: slow, steady uplift from at least 40 Ma primarily due to crustal thickening^10–16^; and rapid, recent surface uplift post 16 Ma as a result of lower lithosphere removal, magmatic thickening or lower crustal flow^17–25^. Currently used palaeoelevation proxies, such as palaeobotany (refs. ^20,26^ and references therein) and stable isotope techniques^14,17–20^, rely on the assumption that the dependence of parameters such as air temperature and humidity upon elevation in the past were the same as the present^6^. However, regional climate change related to surface uplift may account for some signals used to interpret elevation gain^5,27,28^. Palaeoclimate conditions are often not corrected for, resulting in large errors on palaeoelevation estimates of up to a few kilometres^27,28^.

Very few studies have constrained palaeoelevation estimates for the Western Cordillera^6^. However, geological evidence shows that the Jurassic to Early Cretaceous Andean arc initially developed in an extensional tectonic setting, which gradually changed from marine to continental conditions^10,29^. The onset of compressional deformation in the Western Cordillera between 90 and 70 Ma is evident from angular unconformities, intrusive relationships and extensive conglomerate deposition in back-arc regions^13,30^. Deformation and crustal shortening then became diachronous in both the Western and Eastern cordilleras from c. 50–40 Ma^11,31^. At this time the present high Altiplano-Puna was a ~300 km-wide basin close to sea level, which separated the two deformation belts^32,33^. Marked differences in provenance of Late Eocene-Oligocene sediments between basins east and west of the ‘Proto’-Western Cordillera provide evidence relief formation at this time^34^; with further confirmation from facies changes related to uplift dated around ~35 Ma in forearc basins^35^.

Here we utilise published geochemical and isotopic data for Andean arc lavas to constrain a regional surface uplift history for the Western Cordillera. Arc magma compositions are related to the processes of mantle wedge melting, intra-crustal differentiation and crustal assimilation^36,37,38^ It has long been observed that there are links between crustal thickness and certain geochemical parameters of arc lavas^36,38–40^. Increasing crustal thickness of the overriding plate can affect arc systematics by the following: reducing the thickness of the mantle wedge, decreasing wedge corner flow and thus limiting the extent of mantle melting^37,38,41,42^; raising the pressure of magma fractionation at the Moho and hence changing the stability of certain mineral phases^43–45^; and increasing the degree of intra-crustal differentiation and crustal assimilation^36,46,47^. Such links have been utilised to infer crustal thickening in the Central Andes from 40 to 30 Ma^10,30,43^. Similarities between geochemical and isotopic signatures of recent Central Andean lavas and mid-Cenozoic lavas from the Great Basin in western Utah and Nevada have led to the interpretation that an orogenic plateau was present at this time^48^, commonly termed the ‘Nevadaplano’. Recently, regional and global compilations of geochemical parameters (such as Sr/Y and La/Yb) of both arc and continental collision zone magmatism have been calibrated to modern crustal thickness^37,41,43–45,49^. Global arc systematics have also been found to correlate with elevation and, assuming isostatic equilibrium^2^, crustal thickness^47^. Despite these numerous findings, arc geochemistry has not previously been directly calibrated to elevation and used to infer a regional surface uplift history. Using age-corrected Sr and Nd isotope ratios we infer that the Western Cordillera was close to current elevations (4200 ± 516 m) by the Early Miocene. From 23 to 10 Ma, surface uplift propagated southwards through the region of the current volcanic gap and northern SVZ, c. 26–35°S.

## Results

### ^87^Sr/^86^Sr and ^143^Nd/^144^Nd ratios as plateau discriminants

**Fig. 2:**
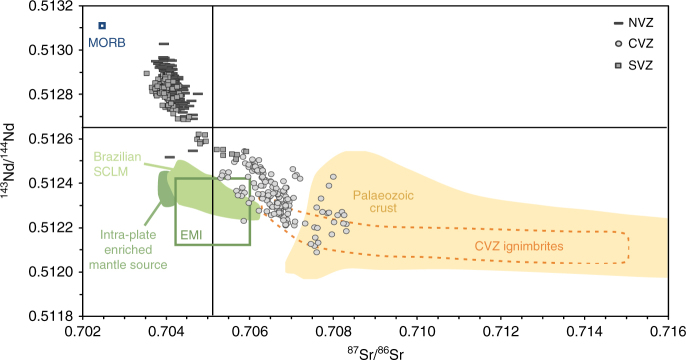
Andean lavas are produced by mixing of depleted mantle melts with radiogenic crust. Sr-Nd radiogenic isotope plot of Quaternary lavas from the NVZ, CVZ and SVZ (compilation from this study, Methods and Supplementary Data 1) and CVZ ignimbrites^73^ in comparison to Palaeozoic continental crust and mantle end members, including Depleted Mantle (Pacific MORB), enriched sub-continental lithospheric mantle (SCLM) and Enriched Mantle I (EMI); from ref. ^56^ and references therein. Andean frontal-arc lavas follow a trend from a depleted mantle source to Palaeozoic crust and are not thought to be influenced by enriched mantle sources^56^

**Fig. 3:**
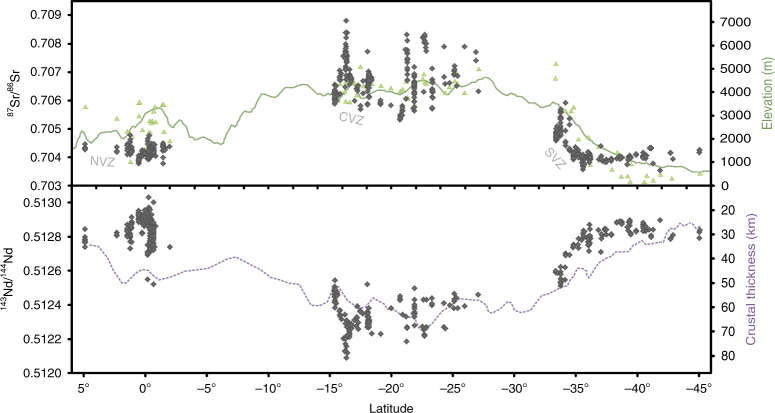
Comparison of whole-rock ^87^Sr/^86^Sr and ^143^Nd/^144^Nd ratios of arc lavas with crustal thickness and elevation. Quaternary whole-rock Sr and Nd isotope ratios of frontal-arc lavas (diamonds, compilation from this study; see Methods and Supplementary Data 1) compared to volcano base elevation (triangles; this study, Methods, and refs. ^69, 74^), arc front mean elevation (100 km-wide swath, GTOPO30 digital elevation model (DEM)^75^) and crustal thickness profiles (five period moving average, data from ref. ^50^, RMS < 3.5 km)

**Fig. 4:**
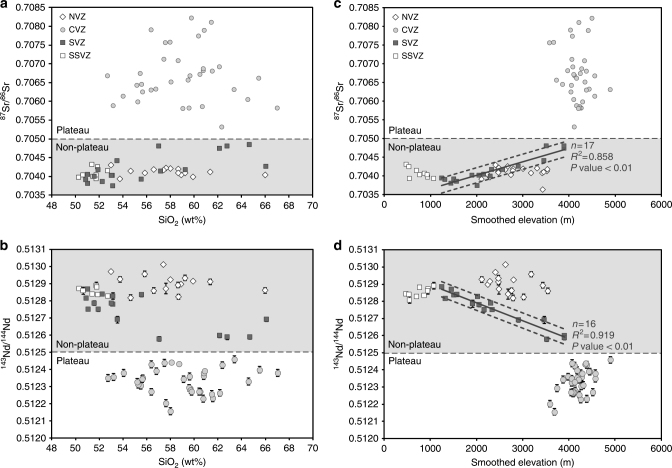
Baseline Sr and Nd isotopes as a plateau discriminant and palaeoelevation proxy. **a**, **b** Baseline isotopic compositions within each volcanic zone vary little with differentiation from basaltic andesite to rhyolite^46^; even the least silicic CVZ rocks are enriched in ^87^Sr/^86^Sr (>0.7050) and depleted in ^143^Nd/^144^Nd (<0.5125). **c**, **d** Baseline isotope compositions compared to smoothed elevation. Elevation smoothed to a radius of 37.5 km was calculated from the Shuttle Radar Topography Mission DEM (SRTM1, pixel resolution 90 m^71^). A radius of 37.5 km is selected as this is half of the maximum crustal thickness in the Andes. Smoothing to this degree filters out non-isostatic, short wave-length topography. 95% confidence intervals are represented as dashed lines (excluding samples south of 38.5°S, see text). Volcano locations are shown on Fig. 1a. Symbols are bigger than the maximum analytical error isotope on isotope data, except where shown. Typical quoted analytical precision on SiO_2_ compositions are ~3% RSD. All data shown here are presented in Supplementary Data 2

^87^Sr/^86^Sr and ^143^Nd/^144^Nd ratios are particularly useful in studying interactions between continental crust and depleted mantle as these reservoirs have a large isotopic contrast (e.g., ref. ^36^, Fig. 2). Qualitative comparisons between ^87^Sr/^86^Sr and ^143^Nd/^144^Nd ratios of Quaternary Andean arc lavas, crustal thickness and present-day topography (Fig. 3) confirm previous findings that radiogenic isotopes can be linked to elevation and crustal thickness. Good correlations between present-day surface elevation and crustal thickness along the Andean arc (Supplementary Fig. 1) support the hypothesis of a dominant isostatic control on regional elevation at the arc. Isotope ratios from CVZ (plateau) arc lavas are clearly distinct from either NVZ or SVZ (non-plateau) arc lavas. For example, NVZ and SVZ lavas have an arithmetic mean (±2 SD) ^87^Sr/^86^Sr ratio of 0.70418 (±0.00036, *n* = 210) and 0.70426 (±0.00098, *n* = 189), respectively, while CVZ lavas have a mean of 0.70671 (±0.00138, *n* = 297). Northern SVZ volcanoes have base elevations over 4000 m (Supplementary Figure 2) and are erupted through crust ~55 km thick^50^ (Fig. 3). Such base elevation and crustal thickness values are comparable to CVZ volcanoes from the Andean Plateau, yet baseline isotope ratios from SVZ centres do not overlap with those from the CVZ. The baseline isotopic signature at each volcanic centre is achieved in zones of melting, assimilation, storage and homogenisation (MASH) at the base of the crust^36,46,51,52^. Rising mantle melts experience assimilation of variable amounts of arc crust^36,46^. CVZ frontal-arc lavas are produced by mixing of mantle melts with 7–37% continental crust^10,46^ (Fig. 2). To minimise the effect of variable mid- to upper-crustal assimilation and allow direct comparison between different volcanic centres, we define the ‘baseline’ isotope composition at each volcanic centre as the least silicic sample (Fig. 4; Methods). CVZ (plateau) lavas have baseline isotopic signatures (^87^Sr/^86^Sr > 0.7050 and ^143^Nd/^144^Nd < 0.5125), which are clearly distinct from NVZ and SVZ (non-plateau) lavas over a similar range in SiO_2_ content^46^ (Fig. 4a, b). Decoupling between isotopic enrichment and major element composition is inferred to reflect prolonged MASH processes in the lower crust^52^. We suggest that this isotopic step change is a result of the tectonic setting varying from a high but narrow arc (northern SVZ and NVZ) to an orogenic plateau (CVZ), as discussed below. Baseline isotope ratios of ^87^Sr/^86^Sr > 0.7050 and ^143^Nd/^144^Nd < 0.5125 discriminate between plateau and non-plateau settings for Andean arc volcanism.

### Correlations between elevation and baseline isotope ratios

Baseline Sr and Nd isotopes of SVZ centres follow strong linear relationships when plotted against both smoothed volcano elevation (Fig. 4c, d) and un-smoothed volcano base elevation (Supplementary Fig. 2). Isotope ratios in the SVZ approach CVZ values from south to north. Sr isotope ratios of southern SVZ (SSVZ; south of 38.5°S) lavas are offset to higher values (Fig. 4d), which can be attributed to numerous large fracture zones on the incoming Nazca plate, which project below the SSVZ^53^. Hydrothermal alteration and serpentinization along these fracture zones increases fluid flux to the mantle wedge, causing a shift to higher ^87^Sr/^86^Sr compositions in the mantle source and in the resultant arc lavas^53^ (Fig. 4c). Therefore, we do not include SSVZ volcanoes in our linear regression analysis.

The Central and Southern Andes have pre-Andean basements mostly comprised of Palaeozoic accreted terranes intruded by Mesozoic arc plutons^36,54^. NVZ arc lavas are erupted through a young, accreted oceanic plateau basement of Mesozoic age^55^. NVZ lavas have more mantle-like isotopic ratios than SVZ lavas regardless of regional elevation or crustal thickness. For this reason, the NVZ is not included in the same linear regression analysis as SVZ lavas (Fig. 4c, d) and we do not attempt a palaeoelevation reconstruction for the NVZ. Basements within the CVZ and SVZ produce comparable Sr- and Nd-isotopic shifts for the same degree of crustal contamination if other factors such as the slab parameter and mantle source are equivalent^36,54^. Geochemical studies indicate the Andean arc front (18–40°S) has tapped a relatively homogenous depleted mantle source since the Jurassic^56^. Back-arc lavas have chemical signatures which indicate a range of mantle sources^29,57^ for this reason we do not include them in our compilation. Therefore, our regional palaeoelevation estimates only apply to the arc front and present Western Cordillera, not to the back-arc regions in the Altiplano-Puna.

### Regional surface uplift history for the Western Cordillera

**Fig. 5:**
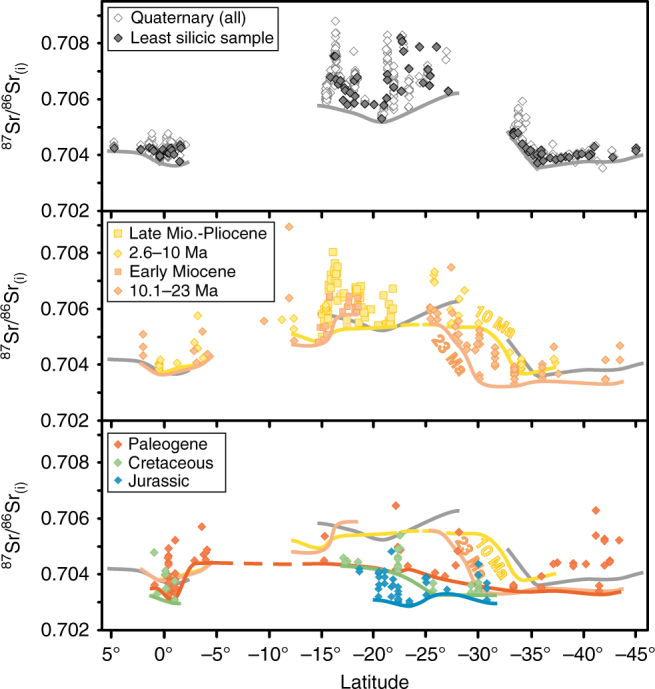
Evolution of Andean arc initial Sr isotope compositions from the Jurassic to present. Age-corrected Sr isotope ratios of arc lavas (compilation of this study, Supplementary Data 3) grouped by age show the gradual increase in ^87^Sr/^86^Sr_(i)_ with time. Baselines are drawn joining minimum values for each age group. Symbols are bigger than the maximum analytical error. For distribution of CVZ analyses versus age, please see Supplementary Fig. 3

We apply both our plateau discriminant and Sr-isotope elevation proxy to age-corrected pre-Quaternary isotope data (^87^Sr/^86^Sr_(i)_ and ^143^Nd/^144^Nd_(i)_; Fig. 5; Supplementary Fig. 3) for the CVZ and SVZ to estimate palaeoelevations of the Jurassic-Pliocene Andean arc (~200–2 Ma). We interpret a baseline ^87^Sr/^86^Sr_(i)_ ratio of >0.7050 and ^143^Nd/^144^Nd_(i)_ ratio of <0.5125 as a ‘plateau signature’. We suggest that the isotopic ‘plateau signature’ corresponds to arc elevations similar to the modern Western Cordillera and CVZ (4200 ± 516 m, mean smoothed elevation of the Western Cordillera along the arc ± 2 SD), but does not correspond to the current width of the entire Andean Plateau. Less radiogenic isotope ratios (^87^Sr/^86^Sr_(i)_ < 0.7050 and ^143^Nd/^144^Nd_(i)_ > 0.5125) are interpreted to correspond to regional elevations according to the linear relationships we have identified for the SVZ (Fig. 4c, d). Due to better data coverage of ^87^Sr/^86^Sr_(i)_ ratios for pre-Quaternary samples we show only our palaeoelevation estimates based on Sr isotope compositions. We divide pre-Quaternary radiogenic isotope data into age intervals selected according to the data density (Fig. 5), which permits two broad groups for the Miocene-Pliocene (23–10 and 10–2 Ma), but only one each for the Paleogene, Cretaceous and Jurassic periods. Jurassic Central Andean lavas have ^87^Sr/^86^Sr_(i)_ ratios analogous to the modern southern SVZ, with baseline initial Sr isotope ratios gradually increasing through the Cretaceous and Paleogene (Fig. 5; Supplementary Figure 3). The largest increase in ^87^Sr/^86^Sr_(i)_ occurs by ~23 Ma (Early Miocene) between 16 and 26°S where baseline initial isotope ratios reach values ^87^Sr/^86^Sr_(i)_ > 0.7050. From 23 to 10 Ma, in the region between 26 and 33°S there is a gradual increase in ^87^Sr/^86^Sr_(i)_ from ~0.7035 to ~0.7050.

## Discussion

**Fig. 6:**
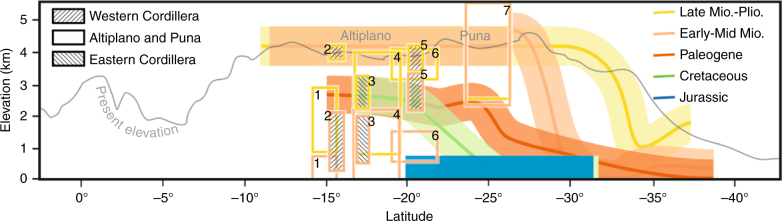
Surface uplift of the Central and Southern Andes from Jurassic to present. Coloured lines are our inferred regional palaeoelevation estimates for the Western Cordillera using our Sr isotope plateau discriminant and SVZ calibration. Past plateau elevation is inferred to be similar to present-day Western Cordillera elevations (4200 ± 516 m, mean CVZ volcano elevation ±2 SD, using 37.5 km smoothing). Non-plateau palaeoelevation estimates have lines set to a thickness representing 95% confidence (±564 m). For general comparison, previously published palaeoelevation estimates are shown in boxes, simplified to fit the same time intervals used in this study (1^20^, 2^59^, 3^19^, 4^17, 18^, 5^76^, 6^21^ and 7^14^)

Figure 6 shows our regional palaeoelevation estimates for the Andean arc and Western Cordillera (coloured lines) compared to previous palaeoelevation estimates for the Western Cordillera, Altiplano-Puna and Eastern Cordillera (boxes). A limitation of our method is it will produce overestimates on palaeoelevation where volcanic suites are not analysed or preserved at the least radiogenic end of the range of compositions. We anticipate this issue may only apply to the Jurassic to Paleogene periods for which data are sparse. Jurassic arc baseline compositions and consequent palaeoelevation estimates are consistent with geological observations of marine sedimentary rocks intercalated with lavas of that age (ref. ^10^ and references therein). However, geological evidence suggests that Central Andean basins remained dominantly marine up until the mid-Cretaceous (91 Ma)^10^, indicating lack of Sr isotope data for this period are causing overestimates in our elevation model (Fig. 6). A marked shift in Central Andean baseline compositions between the Paleogene and Early Miocene indicates an increase in arc elevation of ~2 km. Our results suggest that by 23 Ma, between at least 16 and 26°S, the Andean arc was part of a tectonic plateau, which we suggest attained elevations comparable to that of the modern Western Cordillera (4200 ± 516 m; Fig. 6). Our results do not preclude minor uplift or tilting of the Western Cordillera in the Miocene^58^, as the Western Cordillera could have been at the lower limit of our estimated range at 23 Ma and risen to current elevations since. The width of elevated areas at this time may have been similar to the modern Western Cordillera (~50–100 km). Such widths are much narrower than the modern Andean Plateau (~400 km, which encompasses the Western Cordillera, Altiplano-Puna and Eastern Cordillera), but are wider than either the NVZ or SVZ (mostly <50 km). This conclusion is consistent with sediment providence data and facies changes indicating the presence of a Proto-Western Cordillera by the Late Eocene-Oligocene^34,35^, and also with evidence of eastward propagation of a narrow, early fold-thrust belt into the Eastern Cordillera at ~40 Ma^11,31^. We propose the Western Cordillera was ~2 km higher at 23 Ma than a study from ~15°S^59^, which suggests palaeoelevations of ~2 km by 19 Ma (Fig. 6). Our result predates rapid Late Miocene (10–6 Ma) surface uplift interpreted for the Altiplano to the east^18,20^, but is consistent with sediment provenance data which indicate that the Western Cordillera rose earlier than the Altiplano^34,60^. Our results agree with stable isotope evidence suggesting the south-eastern Puna Plateau was at similar to modern elevations by ~36 Ma^14^ (Fig. 6). Between 23 and 10 Ma we interpret surface uplift of the Western Cordillera to have propagated further south by ~400 km.

Studies on Altiplano palaeoelevation have emphasised large-scale loss of lower lithosphere as a mechanism for generating rapid surface uplift in this region between 10 and 6 Ma^17,18^. Delamination of the lower lithosphere and asthenospheric upwelling results in preferential melting of the most fertile, decompressed or heated mantle^61^. Pliocene-Quaternary back-arc lavas within the Altiplano and Puna have geochemical signatures suggested to be consistent with small-scale delamination or dripping^57,61–63^, which are not present in the frontal arc to the west^10^. Hence, we only calibrate isotope signatures of frontal-arc lavas to elevation and our regional palaeoelevation estimates apply only to the (proto-) Western Cordillera.

We have identified strong linear correlations between baseline Sr and Nd radiogenic isotope compositions of SVZ arc lavas and elevation, and by implication, crustal thickness. Our findings using radiogenic isotope chemistry are complementary to previous studies on relationships between trace element chemistry of arc lavas and crustal thickness^37,41,43,44^. Crustal thickness of the overriding plate in a subduction zone controls arc lava chemistry by some of, or a combination of all, the following processes: mantle wedge thermal structure and melting regime^37,38,41,42^; pressure of magma fractionation at the base of the crust^43–45^; and the degree of crustal assimilation^36,46,47^. Correlations between ^87^Sr/^86^Sr and ^143^Nd/^144^Nd isotopes and SVZ elevation (and crustal thickness) do not preclude either of the first two processes but indicate that there is a relationship between crustal thickness and the degree of crustal assimilation. Therefore, we can indirectly gain an insight on the palaeoelevation and tectonic history of the Central Andes using age-corrected radiogenic isotope data, which is consistent with the geological record for this region.

The slopes of the correlations we have identified here (Fig. 4c, d; Supplementary Fig. 2) cannot be directly applied to other arcs. If correlations between Sr and Nd isotopes and elevation can be found elsewhere, the slope will depend principally upon the isotopic difference between the mantle source and overriding crust. Our method in determining regional elevation may be applied to other arcs as long as all the steps we have laid out here are followed. There must be an active segment of the arc where Quaternary isotope compositions can be directly correlated to present-day elevation. Careful work needs to go into checking arc segments in question to determine if there is reasonable justification to study the elevation (and crustal thickness) control on isotope compositions in isolation. Furthermore, there must be sufficient isotope data available to reliably find the baseline isotope ratio for each volcanic centre. The Central American arc has potential for quantitative relationships between radiogenic isotopes and elevation to be explored further, as qualitative correlations between isotope ratios and crustal thickness have already been found^40^.

The isotopic step change we utilise as a plateau discriminant indicates the relationship between elevation, crustal thickness and isotope composition is not a simple linear trend like that observed for the SVZ (Fig. 4c, d^42^). The isotopic shift between non-plateau and plateau lavas implies more crustal contamination in plateau (CVZ) lavas than would be expected from linear extrapolation of SVZ trends. However, the mechanisms of isotopic enrichment within orogenic plateaux are relatively unknown and we highlight this topic as an area for further research. It is possible that the great width (>400 km) of thick (>60 km) crust across the Andean Plateau raises the geothermal gradient across this broad region rather than along a narrow arc leading to a hot, weak lower crust^7^. Lower crustal heating due to potential asthenosphere upwelling must also be taken into account^61,64^.

Our approach utilises abundant radiogenic isotope data from previous studies of Andean arc geochemistry to provide a regional perspective on the surface uplift and tectonic history of the Andes through time. Calibrating radiogenic isotope compositions of arc lavas to smoothed elevation provides a new indirect palaeoelevation proxy and plateau discriminant that does not rely on palaeoclimate. Miocene (from 23 Ma) to recent Central Andean arc lavas all have a ‘plateau’ isotope signature (^87^Sr/^86^Sr > 0.705 and ^143^Nd/^144^Nd < 0.5125). We suggest that between 16 and 26°S the Western Cordillera attained current elevations (4200 ± 516 m) by 23 Ma. Our results do not preclude minor tilting or uplift of the Western Cordillera during the Early Miocene. We suggest Western Cordillera elevations were reached ~15 Myr before significant surface uplift previously determined for the adjacent Altiplano to the east. During the Early-Mid Miocene, surface uplift propagated southward between ~26 and 35°S.

## Methods

### Geochemical compilation

We have compiled a geochemical database for Andean arc lavas, dated from Jurassic to present day, from 41 previously published studies (see Supplementary References 5,9–48). We have not included ignimbrites, plutonic rocks or back-arc lavas in this database. Jurassic centres that are known to have been erupted underwater and hence have isotope signatures, which are affected by seawater contamination^29^ are not included. The maximum analytical error reported in our compilation is <±0.00007 2*σ* for Sr isotope ratios and ±0.00003 2*σ* for Nd isotope ratios.

*Isotope standards*: ^87^Sr/^86^Sr ratios of each of the two standards (Eimer and Amend, and NBS 987) reported by different laboratories in our compilation are within analytical error so data can be directly compared, except for data published by Francis et al.^65^ and Rogers and Hawkesworth^66^, which have been normalised to 0.70800 (Eimer and Amend) and 0.71025 (NBS 987), respectively. Quaternary ^143^Nd/^144^Nd ratios of reported standards are also within error, except data from Marín-Cerón et al.^67^ and Nyström et al.^68^, which are both normalised to 0.511840 (La Jolla). No other isotope ratios have been normalised. Sr and Nd isotope standards can be obtained from the corresponding author on request. For geochemical compilation references, see Supplementary References 5,9–48.

### Defining baseline isotopic signatures

Data in Fig. 3 (Supplementary Data 2) are isotope ratios of the least silicic sample from each volcanic centre in our compilation. Where major element compositions are not published, we choose the least radiogenic sample for that volcanic centre. We do not include centres with only one reported sample.

### NVZ base elevations

To define base elevations of volcanoes from the NVZ for use in Fig. 3, Supplementary Fig. 1 and Supplementary Fig. 2, we use the methods of Völker et al.^69^ so that results may be compared to the SVZ. The locations of 31 Holocene volcanoes were taken from the ‘volcanoes of the world’ database (Smithsonian Institute^70^). The base elevation of each centre is defined as the lowest elevation value (baseline minimum *b*^69^) of the basal plane. To define the outer boundary and basal plane of each centre, Digital Elevation Models (DEMs) from the Shuttle Radar Topography Mission (SRTM1; 90 m pixel resolution^71^) were compared with satellite images from Google Earth. Each centre is assigned a morphometric classification^72^, ranging from 1 (individual, symmetrical cone/stratovolcano and morphologically well defined) to 4 (complex massif with multiple edifices or calderas, may also be heavily incised). These values give an estimate of the reliability of the measurements, as the outer boundary of morphologically well-defined volcanoes (category 1) can be easily found from a sudden change in gradient, which equates to the change from volcanic edifice to basal plain^69^. Outer boundaries, and hence basal elevation, are much harder to determine for heavily eroded, collapsed or complex centres built upon uneven terrain, which is the case for the majority of centres in the NVZ. DEM images were consistently checked against images of each volcano on Google Earth, so as to better define the baseline using variations in colour of the base rock and changes in degree of vegetation.

### Data availability

All data used in this manuscript are available in Supplementary Data 1–3. Further queries and information requests should be directed to the lead author E.M.S.(erin.scott@durham.ac.uk).

## Electronic supplementary material

Supplementary Information

Description of Additional Supplementary Files

Supplementary Data 1

Supplementary Data 2

Supplementary Data 3
