# Supplementary material for: Andean surface uplift constrained by radiogenic isotopes of arc lavas
Source: Nat Commun. 2018 Mar 6;9:969. doi: 10.1038/s41467-018-03173-4 (PMC5840411; doi:10.1038/s41467-018-03173-4)
Supplement: Supplementary file 1 — Supplementary Information [file 41467_2018_3173_MOESM1_ESM.pdf]

Andean surface uplift constrained by radiogenic isotopes of arc lavas

Erin M. Scott[1]*, Mark B. Allen[1], Colin G. Macpherson[1], Ken J.W. McCaffrey[1], Jon P. Davidson[1], Christopher Saville[1] and Mihai N. Ducea[2,3]

[1] Department of Earth Sciences, Durham University, Durham, DH1 3LE, UK

[2] Department of Geosciences, University of Arizona, Tucson, Arizona 85721, USA

[3] Faculty of Geology and Geophysics, University of Bucharest, 010041 Bucharest, Romania

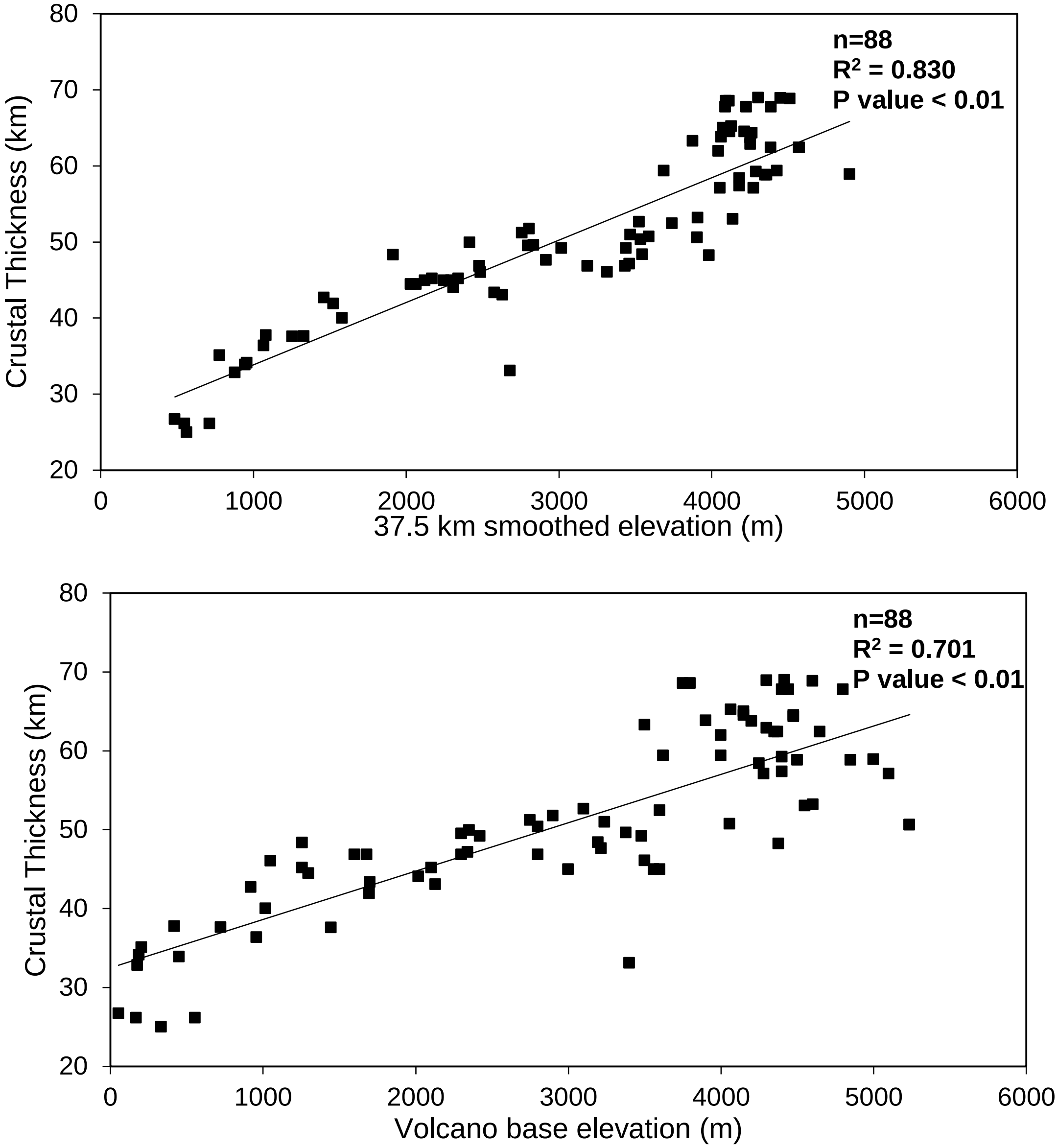

Supplementary Figure 1 | Statistical comparison of crustal thickness and elevation at Andean arc volcanoes. Crustal thickness (ref. 1; RMS <3.5 km) and volcano base elevation (refs. 2,3 and Methods) data are the same as Figure 1. Smoothed elevation (same as Figure 3) was calculated using focal statistics on Shuttle Radar Topography Mission DEMs (SRTM, 90 m pixel resolution; ref. 4). A radius of 37.5 km was selected as this is half of the maximum crustal thickness in the Andean Plateau.

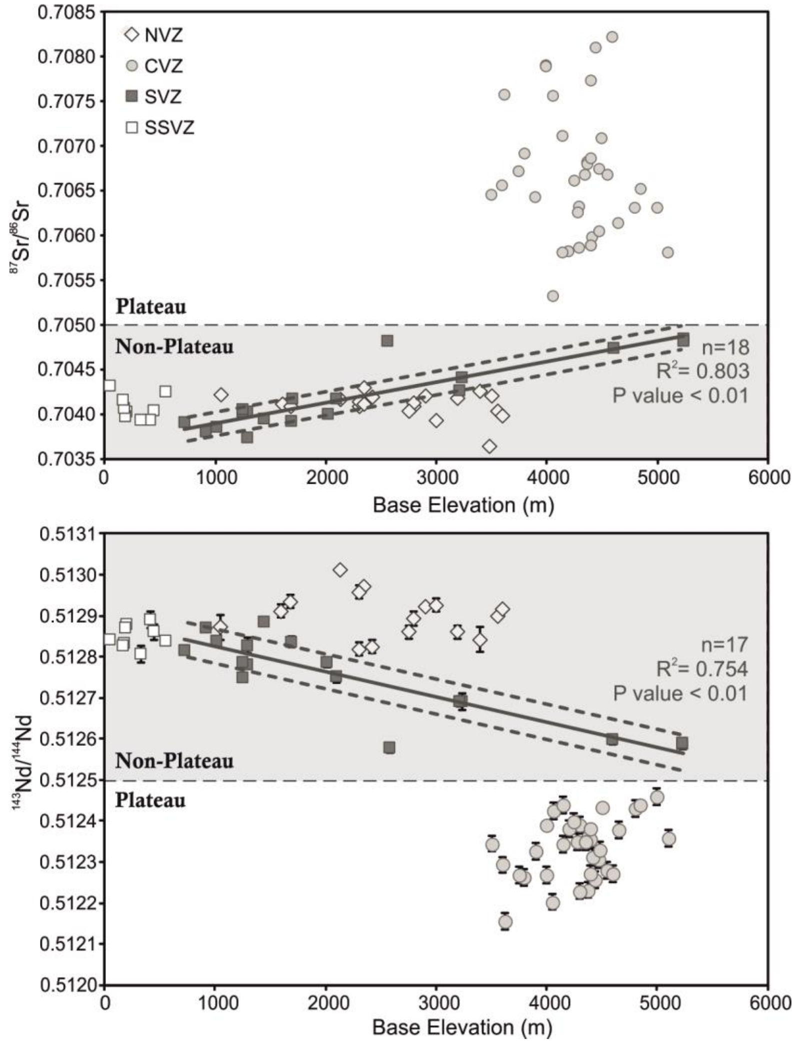

Supplementary Figure 2 | **Baseline Sr- and Nd- isotopes as a plateau discriminant and paleoelevation proxy.** Un-smoothed volcano base elevation data (refs. 2,3 and Methods) are the same as Figure 1. Baseline Sr- and Nd-data are the same as Figure 3. Linear correlations are still strong despite using different methods to obtain volcano elevations. Slopes identified using base elevation data produce paleo-base elevation estimates that are within error of smoothed elevation estimates using calibrations shown in Figure 3. The correlation between $^{143}Nd/^{144}Nd$ and volcano base elevation can be extended to include southern SVZ (south of 38.5°S, SSVZ) centres (n=27, R^2 = 0.825, P value < 0.01).

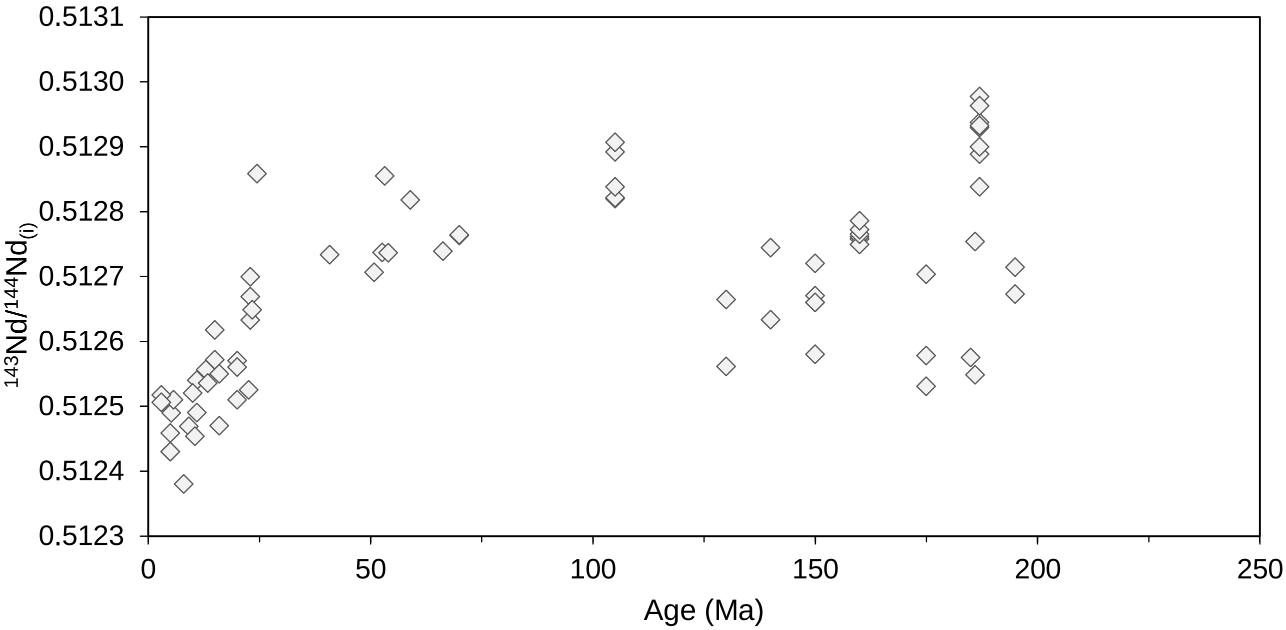

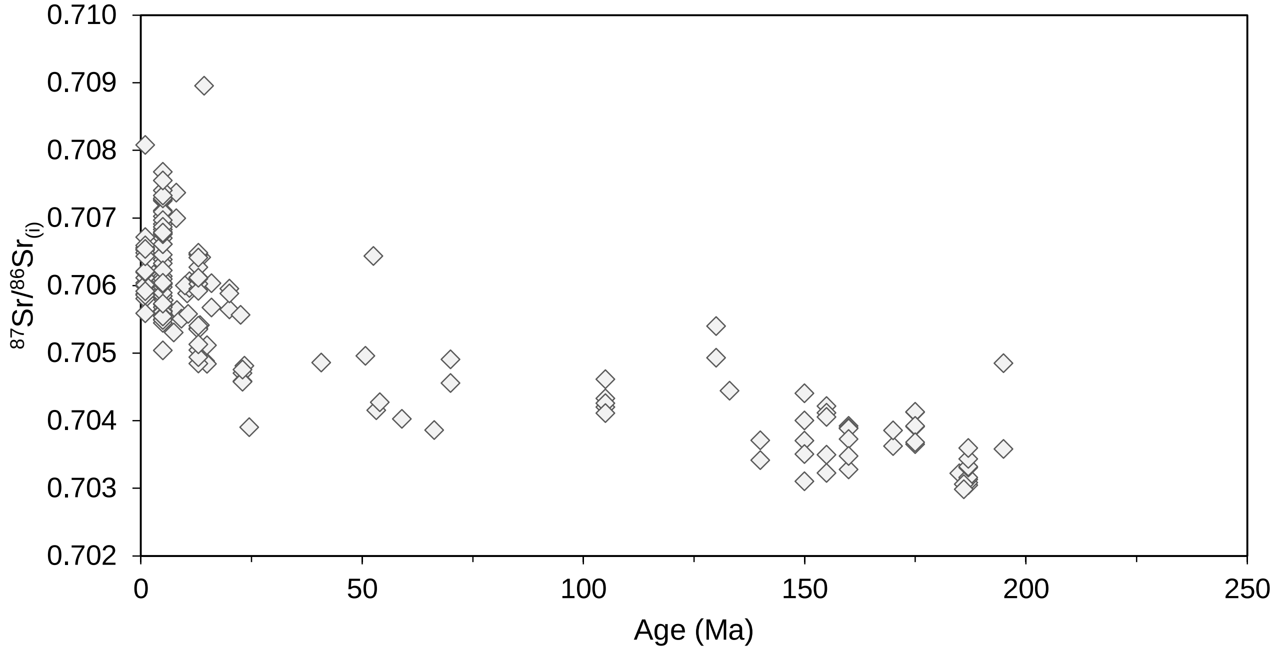

Supplementary Figure 3 | Evolution of age corrected Sr- (upper panel) and Nd- (lower panel) isotope ratios in Central Andean lavas through time. Data are the same as Figure 4 between 12-30° S (Supplementary Table 3), excluding Quaternary data. This plot follows on from Haschke et al.[5] and the review of DeCelles et al.[6], including more recent analyses.

Supplementary References

1. Assumpção, M., Feng, M., Tassara, A. & Julià, J. Models of crustal thickness for South America from seismic refraction, receiver functions and surface wave tomography. *Tectonophysics* **609,** 82–96 (2013).
2. de Silva, S. L. & Francis, P. W. *Volcanoes of the central Andes*. (Springer-Verlag, 1991).
3. Völker, D., Kutterolf, S. & Wehrmann, H. Comparative mass balance of volcanic edifices at the southern volcanic zone of the Andes between 33°S and 46°S. *J. Volcanol. Geotherm. Res.* **205,** 114–129 (2011).
4. Farr, T. G. *et al.* The Shuttle Radar Topography Mission. *Rev. Geophys.* **45,** RG2004 (2007).
5. Haschke, M., Siebel, W., Günther, A. & Scheuber, E. Repeated crustal thickening and recycling during the Andean orogeny in north Chile (21°–26°S). *J. Geophys. Res. Solid Earth* **107,** ECV 1-18 (2002).
6. DeCelles, P. G., Ducea, M. N., Kapp, P. & Zandt, G. Cyclicity in Cordilleran orogenic systems. *Nat. Geosci.* **2,** 251–257 (2009).
7. Global Volcanism Program. *Volcanoes of the World, v. 4.5.0* (2013). Available at: http://volcano.si.edu/. (Accessed: 6th September 2016)
8. Grosse, P., Vries, B. van W. de, Petrinovic, I. A., Euillades, P. A. & Alvarado, G. E. Morphometry and evolution of arc volcanoes. *Geology* **37,** 651–654 (2009).
9. Francis, P. W., Moorbath, S. & Thorpe, R. S. Strontium isotope data for Recent andesites in Ecuador and North Chile. *Earth Planet. Sci. Lett.* **37,** 197–202 (1977).
10. Rogers, G. & Hawkesworth, C. J. A geochemical traverse across the North Chilean Andes: evidence for crust generation from the mantle wedge. *Earth Planet. Sci. Lett.* **91,** 271–285 (1989).
11. Marín-Cerón, M. I., Moriguti, T., Makishima, A. & Nakamura, E. Slab decarbonation and CO2 recycling in the Southwestern Colombian volcanic arc. *Geochim. Cosmochim. Acta* **74,** 1104–1121 (2010).
12. Nyström, J. O., Vergara, M., Morata, D. & Levi, B. Tertiary volcanism during extension in the Andean foothills of central Chile (33°15′–33°45′S). *Geol. Soc. Am. Bull.* **115,** 1523–1537 (2003).

13. Allibon, J. *et al.* The contribution of the young Cretaceous Caribbean Oceanic Plateau to the genesis of late Cretaceous arc magmatism in the Cordillera Occidental of Ecuador. *J. South Am. Earth Sci.* **26,** 355–368 (2008).
14. Béguelin, P., Chiaradia, M., Beate, B. & Spikings, R. The Yanaurcu volcano (Western Cordillera, Ecuador): A field, petrographic, geochemical, isotopic and geochronological study. *Lithos* **218–219,** 37–53 (2015).
15. Bissig, T., Ullrich, T. D., Tosdal, R. M., Friedman, R. & Ebert, S. The time-space distribution of Eocene to Miocene magmatism in the central Peruvian polymetallic province and its metallogenetic implications. *J. South Am. Earth Sci.* **26,** 16–35 (2008).
16. Bissig, T. & Tosdal, R. M. Petrogenetic and Metallogenetic Relationships in the Eastern Cordillera Occidental of Central Peru. *J. Geol.* **117,** 499–518 (2009).
17. Bryant, J. A., Yogodzinski, G. M., Hall, M. L., Lewicki, J. L. & Bailey, D. G. Geochemical Constraints on the Origin of Volcanic Rocks from the Andean Northern Volcanic Zone, Ecuador. *J. Petrol.* **47,** 1147–1175 (2006).
18. Chiaradia, M. & Fontboté, L. Radiogenic Lead Signatures in Au-Rich Volcanic-Hosted Massive Sulfide Ores and Associated Volcanic Rocks of the Early Tertiary Macuchi Island Arc(Western Cordillera of Ecuador). *Econ. Geol.* **96,** 1361–1378 (2001).
19. Chiaradia, M., Fontboté, L. & Beate, B. Cenozoic continental arc magmatism and associated mineralization in Ecuador. *Miner. Deposita* **39,** 204–222 (2004).
20. Chiaradia, M. Adakite-like magmas from fractional crystallization and melting-assimilation of mafic lower crust (Eocene Macuchi arc, Western Cordillera, Ecuador). *Chem. Geol.* **265,** 468–487 (2009).
21. Chiaradia, M., Müntener, O. & Beate, B. Enriched Basaltic Andesites from Mid-crustal Fractional Crystallization, Recharge, and Assimilation (Pilavo Volcano, Western Cordillera of Ecuador). *J. Petrol.* **52,** 1107–1141 (2011).

22. Chiaradia, M., Müntener, O. & Beate, B. Quaternary Sanukitoid-like Andesites Generated by Intracrustal Processes (Chacana Caldera Complex, Ecuador): Implications for Archean Sanukitoids. *J. Petrol.* **55,** 769–802 (2014).
23. Entenmann, J. Magmatic evolution of the Nevados de Payachata complex and the petrogenesis of basaltic andesites in the Central Volcanic Zone of northern Chile. (Universitat Mainz, 1994).
24. Freymuth, H., Brandmeier, M. & Wörner, G. The origin and crust/mantle mass balance of Central Andean ignimbrite magmatism constrained by oxygen and strontium isotopes and erupted volumes. *Contrib. Mineral. Petrol.* **169,** 58 (2015).
25. Futa, K. & Stern, C. R. Sr and Nd isotopic and trace element compositions of Quaternary volcanic centers of the Southern Andes. *Earth Planet. Sci. Lett.* **88,** 253–262 (1988).
26. Garrison, J. M., Davidson, J. P., Hall, M. & Mothes, P. Geochemistry and Petrology of the Most Recent Deposits from Cotopaxi Volcano, Northern Volcanic Zone, Ecuador. *J. Petrol.* **52,** 1641–1678 (2011).
27. Gil-Rodriguez, J. Petrology of the Betulia Igneous Complex, Cauca, Colombia. *J. South Am. Earth Sci.* **56,** 339–356 (2014).
28. Hickey, R. L., Frey, F. A., Gerlach, D. C. & Lopez-Escobar, L. Multiple sources for basaltic arc rocks from the southern volcanic zone of the Andes (34°–41°S): Trace element and isotopic evidence for contributions from subducted oceanic crust, mantle, and continental crust. *J. Geophys. Res. Solid Earth* **91,** 5963–5983 (1986).
29. Hildreth, W. & Moorbath, S. Crustal contributions to arc magmatism in the Andes of Central Chile. *Contrib. Mineral. Petrol.* **98,** 455–489 (1988).
30. Jacques, G. *et al.* Across-arc geochemical variations in the Southern Volcanic Zone, Chile (34.5–38.0°S): Constraints on mantle wedge and slab input compositions. *Geochim. Cosmochim. Acta* **123,** 218–243 (2013).
31. Jacques, G. *et al.* Geochemical variations in the Central Southern Volcanic Zone, Chile (38–43°S): The role of fluids in generating arc magmas. *Chem. Geol.* **371,** 27–45 (2014).

32. James, D. E. & Murcia, L. A. Crustal contamination in northern Andean volcanics. *J. Geol. Soc.* **141,** 823–830 (1984).

33. Kay, S. M., Maksaev, V., Moscoso, R., Mpodozis, C. & Nasi, C. Probing the evolving Andean Lithosphere: Mid-Late Tertiary magmatism in Chile (29°–30°30'S) over the modern zone of subhorizontal subduction. *J. Geophys. Res. Solid Earth* **92,** 6173–6189 (1987).

34. Kay, S. M., Mpodozis, C., Ramos, V. A. & Munizaga, F. Magma source variations for mid–late Tertiary magmatic rocks associated with a shallowing subduction zone and a thickening crust in the central Andes (28 to 33°S). *Geol. Soc. Am. Spec. Pap.* **265,** 113–138 (1991).

35. Kay, S. M., Mpodozis, C., Tittler, A. & Cornejo, P. Tertiary Magmatic Evolution of the Maricunga Mineral Belt in Chile. *Int. Geol. Rev.* **36,** 1079–1112 (1994).

36. Kay, S. M., Burns, W. M., Copeland, P. & Mancilla, O. Upper Cretaceous to Holocene magmatism and evidence for transient Miocene shallowing of the Andean subduction zone under the northern Neuquén Basin. *Geol. Soc. Am. Spec. Pap.* **407,** 19–60 (2006).

37. Kramer, W. *et al.* Geochemical and isotopic characteristics and evolution of the Jurassic volcanic arc between Arica (18°30'S) and Tocopilla (22°S), North Chilean Coastal Cordillera. *Chem. Erde - Geochem.* **65,** 47–78 (2005).

38. Lucassen, F. *et al.* Nd, Pb, and Sr isotope composition of juvenile magmatism in the Mesozoic large magmatic province of northern Chile (18–27°S): indications for a uniform subarc mantle. *Contrib. Mineral. Petrol.* **152,** 571–589 (2006).

39. Mamani, M., Tassara, A. & Wörner, G. Composition and structural control of crustal domains in the central Andes. *Geochem. Geophys. Geosystems* **9,** Q03006 (2008).

40. Maydagán, L. *et al.* Petrology of the Miocene igneous rocks in the Altar region, main Cordillera of San Juan, Argentina. A geodynamic model within the context of the Andean flat-slab segment and metallogenesis. *J. South Am. Earth Sci.* **32,** 30–48 (2011).

41. Morata, D. & Aguirre, L. Extensional Lower Cretaceous volcanism in the Coastal Range (29°20′–30°S), Chile: geochemistry and petrogenesis. *J. South Am. Earth Sci.* **16,** 459–476 (2003).

42. Muñoz, J. *et al.* The relation of the mid-Tertiary coastal magmatic belt in south-central Chile to the late Oligocene increase in plate convergence rate. *Rev. Geológica Chile* **27,** 177–203 (2000).

43. Rossel, P. *et al.* The Early Andean subduction system as an analog to island arcs: Evidence from across-arc geochemical variations in northern Chile. *Lithos* **179,** 211–230 (2013).

44. Stern, C. R., Skewes, M. A. & Arévalo, A. Magmatic Evolution of the Giant El Teniente Cu–Mo Deposit, Central Chile. *J. Petrol.* **52,** 1591–1617 (2011).

45. Trumbull, R. B. *et al.* Evidence for Late Miocene to Recent contamination of arc andesites by crustal melts in the Chilean Andes (25–26°S) and its geodynamic implications. *J. South Am. Earth Sci.* **12,** 135–155 (1999).

46. Vatin-Pérignon, N. *et al.* Andean geodynamics Geodynamic interpretations of plate subduction in the northernmost part of the Central Volcanic Zone from the geochemical evolution and quantification of the crustal contamination of the Nevado Solimana volcano, southern Peru. *Tectonophysics* **205,** 329–355 (1992).

47. Vergara, M., Morata, D., Hickey-Vargas, R., López-Escobar, L. & Beccar, I. Cenozoic tholeiitic volcanism in the Colbún area, Linares Precordillera, central Chile (35°S-36°S). *Rev. Geológica Chile* **26,** 23–41 (1999).

48. Wittman, S. Die fazielle Entwicklung des jurassischen '"back arc"'-Beckens in der Kustenkordillere von N-Chile zwischen Zapiga und Arica. *Terra Nostra* **99,** 295–296 (1999).
