# Supplementary material for: Andean surface uplift constrained by radiogenic isotopes of arc lavas
Source: Nat Commun. 2018 Mar 6;9:969. doi: 10.1038/s41467-018-03173-4 (PMC5840411; doi:10.1038/s41467-018-03173-4)
Supplement: Supplementary file 2 — Description of Additional Supplementary Files [file 41467_2018_3173_MOESM2_ESM.pdf]

Description of Additional Supplementary Files

File Name: Supplementary Data 1
Description: Compilation of Quaternary Sr- and Nd- radiogenic isotope compositions of frontal arc lavas.

File Name: Supplementary Data 2
Description: Andean volcano elevations, crustal thicknesses and baseline isotope compositions.

File Name: Supplementary Data 3
Description: Compilation of age corrected Sr- and Nd- radiogenic isotope compositions of JurassicNeogene frontal arc lavas.
